# Plasma and Serum Lipidomics of Healthy White Adults Shows Characteristic Profiles by Subjects’ Gender and Age

**DOI:** 10.1371/journal.pone.0091806

**Published:** 2014-03-14

**Authors:** Masaki Ishikawa, Keiko Maekawa, Kosuke Saito, Yuya Senoo, Masayo Urata, Mayumi Murayama, Yoko Tajima, Yuji Kumagai, Yoshiro Saito

**Affiliations:** 1 Division of Medicinal Safety Science and Disease Metabolome Project, National Institute of Health Sciences, Setagaya, Tokyo, Japan; 2 Clinical Trial Center, Kitasato University East Hospital, Sagamihara, Kanagawa, Japan; INRCA, Italy

## Abstract

Blood is a commonly used biofluid for biomarker discovery. Although blood lipid metabolites are considered to be potential biomarker candidates, their fundamental properties are not well characterized. We aimed to (1) investigate the matrix type (serum vs. plasma) that may be preferable for lipid biomarker exploration, (2) elucidate age- and gender-associated differences in lipid metabolite levels, and (3) examine the stability of lipid metabolites in matrix samples subjected to repeated freeze-thaw cycles. Using liquid chromatography-mass spectrometry, we performed lipidomic analyses for fasting plasma and serum samples for four groups (15 subjects/group) of young and elderly (25–34 and 55–64 years old, respectively) males and females and for an additional aliquot of samples from young males, which were subjected to repeated freeze-thaw cycles. Lysophosphatidylcholine and diacylglycerol levels were higher in serum than in plasma samples, suggesting that the clotting process influences serum lipid metabolite levels. Gender-associated differences highlighted that the levels of many sphingomyelin species were significantly higher in females than in males, irrespective of age and matrix (plasma and serum). Age-associated differences were more prominent in females than in males, and in both matrices, levels of many triacylglycerols were significantly higher in elderly females than in young females. Plasma and serum levels of most lipid metabolites were reduced by freeze-thawing. Our results indicate that plasma is an optimal matrix for exploring lipid biomarkers because it represents the original properties of an individual’s blood sample. In addition, the levels of some blood lipid species of healthy adults showed gender- and age-associated differences; thus, this should be considered during biomarker exploration and its application in diagnostics. Our fundamental findings on sample selection and handling procedures for measuring blood lipid metabolites is important for ensuring the quality of biomarkers identified and its qualification process.

## Introduction

Metabolomics is one of the “omics” platforms for analyzing comprehensive profiles of small molecule metabolites in cells, tissues, or biofluids such as blood and urine. Metabolomics provides a useful tool to analyze metabolite levels in physiological and biological states, and is therefore applied to explore biomarkers for disease diagnosis [1]–[4], and drug responses and toxicity [5]–[8]. Various metabolomic approaches are used, and among these, lipidomics includes the comprehensive analysis of lipid metabolites [9], [10]. Lipid metabolites are not only components of cell membranes but are also involved in signal transduction [11], [12]. Lipid metabolites are therefore considered potential biomarker candidates for disease diagnosis and drug responses. Indeed, recent lipidomic studies have shown that lipid metabolites such as eicosanoids and sphingolipids are biomarker candidates for cardiovascular events [1], traumatic brain injury [13], Alzheimer’s disease [3], [14], type 2 diabetes [15], and depression [16].

Blood is a commonly used biofluid for biomarker discovery because it is a “data-rich” source containing several thousands of hydrophilic and hydrophobic metabolites [17] that likely reflect many complex biological process in the body. In addition, collection of blood samples is a minimally invasive procedure as compared with collection of tissue samples by biopsy. Serum and plasma are two distinct matrices separated from blood after phlebotomy. Serum is prepared from whole blood following a clotting process. Plasma is obtained from whole blood in the presence of an anticoagulant, so that coagulation factors are not activated and thus no blood clot is formed. Because of the differences in the preparation of the two blood matrices, metabolite levels are expected to differ between plasma and serum. Indeed, previous studies, focusing primarily on hydrophilic metabolites, have found differences in the metabolite profiles of plasma and serum, and discussed the advantage of each matrix for metabolite analysis [18]–[21]. As for lipid (hydrophobic) metabolites, we recently investigated the different levels of these molecules in plasma and serum by using non-fasting blood samples from healthy human subjects [21]. In that study, we found that the levels of thromboxane B_2_ (TXB_2_), 12-hydroxy-eicosatetraenoic acid (12-HETE), and 12-hydroxy-eicosapentaenoic acid (12-HEPE) tended to be higher in serum than in plasma, suggesting their release from activated platelets by the clotting process. In the non-fasted condition, however, relatively large inter-individual differences were observed for lipid metabolites levels in blood. On the other hand, matrix-associated differences in lipid metabolite levels remain to be evaluated in fasted subjects. Such data would be valuable because fasting blood is commonly used for biomarker discovery studies and dietary factors are known to affect lipid metabolite levels in blood [22].

To date, potential confounding factors that may affect lipid metabolite levels in the blood for biomarker exploration studies have not been thoroughly evaluated. Although it has been reported that the levels of lipid metabolites such as sphingomyelins (SMs) and phosphatidylcholines (PCs) in blood differ between genders [23], [24], comprehensive data are lacking, especially on the exact molecular species and the extent of differences in their levels among samples derived from subjects from various backgrounds. In order to prevent false-positive or false-negative results in biomarker discovery, gender- and age-associated differences in the basal levels of individual lipid species should be analyzed in advance. Furthermore, it is important to determine an optimal matrix for analyzing lipid biomarkers and to clarify the stability of metabolites subjected to various sample handling and processing procedures. In this study, to obtain fundamental information on lipid metabolites in blood, we aimed to (1) investigate which of the two matrices, plasma or serum, is more suitable for lipid analysis, (2) elucidate gender- and age-associated differences in basal lipid metabolite levels, and (3) examine the stability of lipid metabolites following repeated freeze-thaw cycles of sample matrices. Toward these aims, we performed a lipidomic analysis by using liquid chromatography-mass spectrometry (LC-MS) and liquid chromatography-tandem mass spectrometry (LC-MS/MS) in fasting human plasma and serum samples for four groups (15 subjects/group) consisting of young (25–34 years old) and elderly (55–64 years old) subjects of both genders, and in repeatedly frozen and thawed plasma and serum samples from young male subjects. Because phosphoglycerolipids (PLs) and sphingolipids (SLs) have different physiological functions depending on their classes and fatty acid composition, we identified the exact species of each metabolite to understand gender- and age-associated differences in their levels.

## Materials and Methods

### Collection of Human Blood and Preparation of Plasma and Serum

Blood samples from healthy adults were purchased from PromedDX (Norton, MA). The samples were collected after obtaining written informed consent from all subjects. The ethics committee of the National Institute of Health Sciences authorized PromedDX as a validated provider of blood samples and exempted us from the committee’s approval for use of the purchased blood samples. Venous blood was collected from 60 white subjects on the morning after fasting for 14 h. Participants were divided into four groups of 15 subjects each: young males (25–33 years old), elderly males (55–64 years old), young females (25–34 years old), and elderly females (55–63 years old) (Table 1). Fresh blood from each individual was collected and simultaneously drawn into 10 ml Vacutainer Serum Separator Tubes with a clot activator for serum and 10 ml Vacutainer Plasma Separator Tubes containing K_2_-EDTA for plasma separation. Vacutainer tubes were purchased from Becton Dickinson (Franklin Lakes, NJ). Samples were centrifuged according to the manufacturer’s instructions, and serum and plasma were separated within 2 h after collection of blood samples. The plasma and serum samples were immediately frozen and stored at −80°C. After shipment with dry ice from PromedDX, all frozen samples were thawed once on ice and divided into small aliquots before storing at −80°C until lipid extraction.

**Table 1:** Subject information (fasted white subjects, n = 15 in each group).

Group	Young male (YM)	Young female (YF)	Elderly male (EM)	Elderly female (EF)
Age range (yr)	25–33 (median 29)	25–34 (median 28)	55–64 (median 59)	55–63 (median 59)
Height (cm)	154.9–185.4 (median 172.7)	149.9–182.9 (median 162.6)†	165.1–190.5 (median 177.8)††	152.4–175.3 (median 162.6)†
Weight (kg)	52.2–113.9 (median 78.0)	59.9–147.4 (median 93.4)	63.5–116.1 (median 75.8)	62.6–114.3 (median 90.7)
BMI [kg/m^2^]	18.0–36.6 (median 26.2)	24.9–49.7 (median 35.4)†	19.5–34.9 (median 24.5)	26.1–43.3 (median 32.7)†

(BMI, body mass index).

† Heights and BMIs are significantly different between males and females of corresponding age groups (*p*<0.05 by Man-Whitney *U*-test).

†† Heights of elderly males are significantly higher than those of young males (*p*<0.05 by Mann-Whitney *U*-test).

### Extraction and Measurements of Lipid Metabolites

Lipid extraction and measurement of lipid metabolites by LC-MS(/MS) was performed as reported previously [21]. In brief, small aliquots of frozen plasma and serum were thawed on ice for 2 h. Our normal samples were thus frozen and thawed twice in total, including the dispensing process described above. Lipid metabolites were extracted from 100 µl of plasma or serum by using the method described by Bligh and Dyer (BD) [25] with a few modifications [21]. Lower organic layers were measured by ultra-performance liquid chromatography-time of flight mass spectrometry (UPLC-TOFMS; LCT Premier XE; Waters Micro-mass, Waters, Milford, MA) for analysis of phosphoglycerolipids (PLs), sphingolipids (SLs), and neutral lipids (NLs). To distinguish alkenylacyl and alkyl PL species with the same exact mass, a small aliquot of each BD sample was acid-hydrolyzed [26] and analyzed by UPLC-TOFMS. Upper aqueous layers were subjected to solid extraction to obtain polyunsaturated fatty acids (PUFAs) and their oxidative fatty acids (oxFAs), and then measured by UPLC-MS/MS using a 5500QTRAP quadrupole-linear ion trap hybrid mass spectrometer (AB Sciex, Framingham, MA) interfaced with an ACQUITY UPLC System (Waters, Milford, MA). Structural analysis of PLs and SLs was performed by LC-Fourier Transform Mass Spectrometry (LC-FTMS; LTQ Orbitrap XL, Thermo Fisher Scientific, Waltham, MA) as previously described [26], with a few modifications. Data-dependent MS^3^ analysis was performed in the positive-ion mode to identify the long chain base of ceramides and cerebrosides.

### Effect of Freeze-thawing on the Stability of Lipid Metabolites

To investigate the stability of lipid metabolites in the plasma and serum, we performed 8 additional freeze-thaw cycles (10 cycles in total) in plasma and serum samples of young males. Frozen plasma and serum samples were thawed on ice for 2 h and then re-frozen at −80°C for 30 min. After 10 cycles of freeze-thawing, lipid metabolites were extracted from the samples and analyzed by UPLC-TOFMS and UPLC-MS/MS as described above for normal samples.

### Data Processing

UPLC-TOFMS data were processed using the 2DICAL software (Mitsui Knowledge Industry, Tokyo, Japan) [27]. The extracted ion peaks were normalized using internal standards (ISs). Metabolites eluted from 0.1 to 38.0 min (PLs, SLs, diacylglycerol [DG], and cholesterol [Ch]), and from 37.5 to 60 min (cholestryl ester [ChE], coenzyme Q10 [CoQ10], and triacylglycerol [TG]) by UPLC were separately normalized to 1,2-dipalmitoyl-[^2^H_6_]-*sn*-glycero-3-phosphocholine (16∶0–16∶0 PC-d6; Larodan Fine Chemicals, Malmo, Sweden) and 1,2-dioctanoyl-3-linoleoyl-*sn*-glycerol (8∶0–8∶0–18∶2 TG, Larodan), respectively. Data for PUFAs and oxFAs from UPLC-MS/MS were processed using the MultiQuant™ Software (Version 2.1, AB Sciex, Framingham, MA). The integrated peak area of each metabolite was normalized to deuterated leukotriene B4 (LTB_4_-d4, Cayman Chemical, Ann Arbor, MI).

### Statistical Analysis

The data were analyzed statistically using the Wilcoxon matched-pairs signed-rank tests for comparison of serum and plasma levels for each metabolite for the same group of subjects, and the Mann-Whitney *U*-test test for comparison of metabolite levels among the four groups. Statistical analyses were carried out using R statistical environment (http://r-project.org/) software. Differences with *p* values of less than 0.05 were considered statistically significant.

## Results

### Lipid Metabolite Profiles in Human Blood Samples

Lipidomic analyses were performed for both plasma and serum samples collected from 60 white subjects, and 253 lipid metabolites were thus identified. The metabolites included nine lysophosphatidylcholines (lysoPCs), 34 phosphatidylcholines (PCs), 20 ether-type PCs, two lysophosphatidylethanolamines (lysoPEs), nine phosphatidylethanolamines (PEs), nine ether-type PEs, eight phosphatidylinositols (PIs), 22 sphingomyelins (SMs), seven Cers, eight cerebrosides (CBs), one Ch, 12 ChEs, one CoQ10, seven DGs, 79 TGs (Table S1), three free PUFAs, and 22 oxFAs (Table S2).

### Differences in Lipid Metabolite Profiles between Plasma and Serum Samples

**Figure 1:**
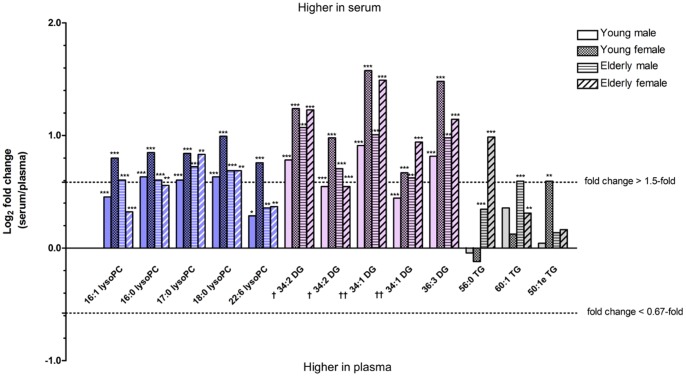
Differences in lipid metabolite levels in human blood samples between plasma and serum. Lipid metabolites with marked fold changes (serum/plasma >1.5 or <0.67) and *p*<0.01 values between plasma and serum in any of the four subject groups are plotted for each group. Statistical significance was determined by Wilcoxon matched-pairs signed-rank test (*p<0.05, **p<0.01, ***p<0.001). Some metabolites (^†, ††^) with same exact mass were eluted with different retention time (Table S1) and therefore seem to be different molecular species of DG. LysoPC, lysophosphatidylcholine; DG, diacylglycerol; TG, triacylglycerol.

**Figure 2:**
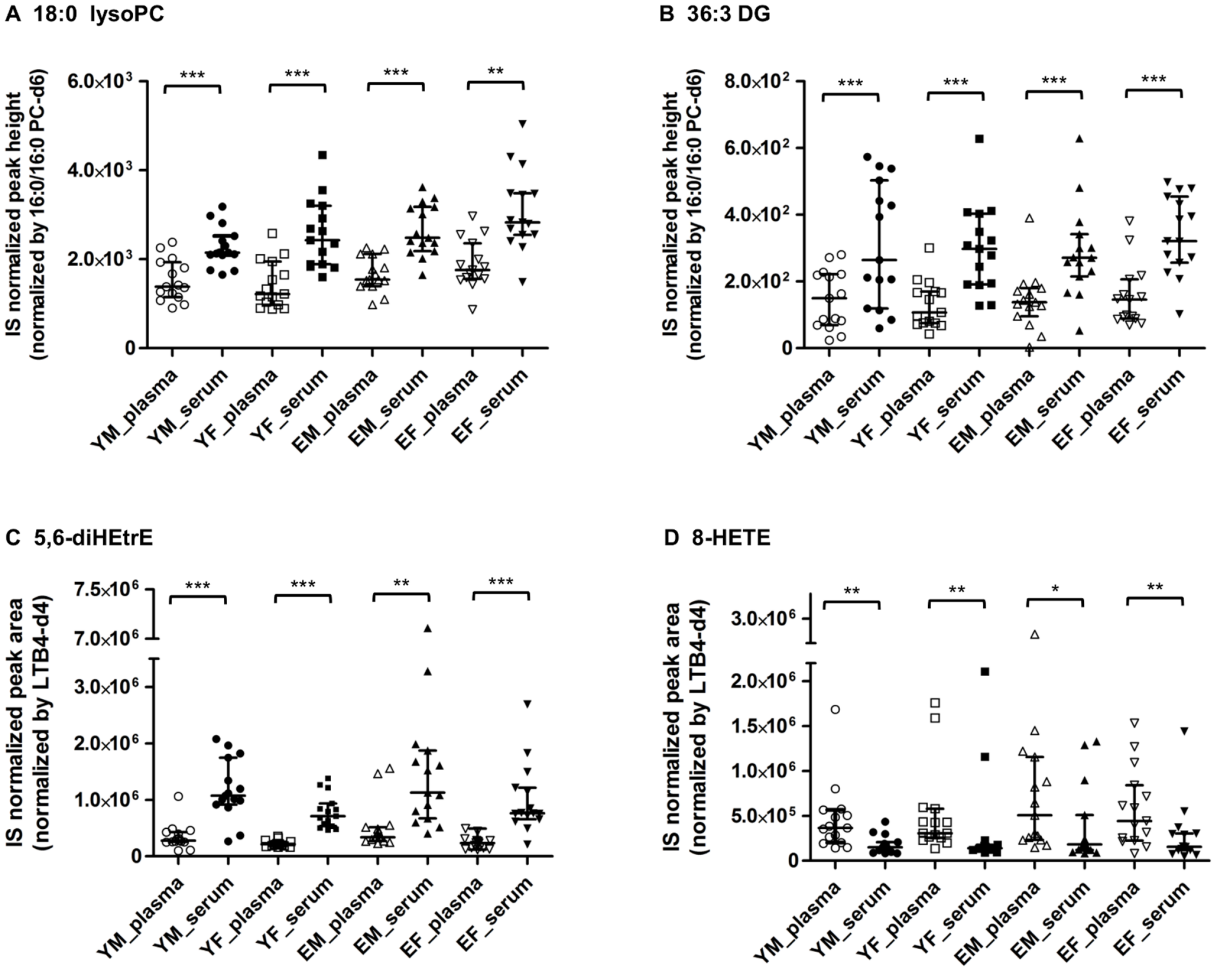
Levels of representative lipid metabolites with significant matrix-associated differences. (A) 18∶0 lysoPC, (B) 36∶3 DG, (C) 5,6-diHEtrE, (D) 8-HETE. The graph shows medians and interquartile ranges. Statistical significance was determined by Wilcoxon matched-pairs signed-rank test (*p<0.05, **p<0.01, ***p<0.001). YM, young male; YF, young female; EM, elderly male; EF, elderly female. LysoPC, lysophosphatidylcholine; DG, diacylglycerol; diHEtrE, dihydroxyeicosatrienoic acid; HETE, hydroxyeicosatetraenoic acid.

Matrix choice is a fundamental consideration for biomarker exploration in blood. We thus first examined the differences in lipid metabolite profiles of plasma and serum in all the groups. Of 253 metabolites, significant differences in plasma and serum levels of 34 metabolites were observed in young males, 82 in young females, 107 in elderly males, and 74 in elderly females (*p*<0.05; Table S3). Levels of PLs and NLs with marked fold changes (serum/plasma ratio >1.5) and lower *p*-values (*p*<0.01) in any of the four groups are shown in Figure 1. Representative species with matrix-associated differences (18∶0 lysoPC and 36∶3 DG) are depicted as scatter plots in Figures 2A and 2B, respectively.

Of the PUFAs and oxFAs, the levels of arachidonic acid (AA), 12-hydroxyheptadecatrienoic acid (12-HHT), 12-hydroxyeicosatetraenoic acid (12-HETE), 5,6-dihydroxyeicosatrienoic acid (5,6-diHETrE), eicosapentaenoic acid, and 4-hydroxyldocosahexaenoic acid (4-HDoHE) were significantly higher in serum than in plasma, whereas four metabolites (8-HETE, 15-HETE, 10-HDoHE, and 20-HDoHE) were present at significantly lower levels in serum than in plasma (*p*<0.05; Table 2) in all the four groups. In particular, the levels of 5,6-diHETrE (Figure 2C) were more than 3.2-fold higher in serum than in plasma. In contrast, the levels of 8-HETE (Figure 2D) in serum were less than half of those in plasma.

**Table 2:** PUFA and oxFA levels showing >1.5 or 0.67-fold changes and *p*<0.01 values between plasma and serum samples of either male or female subjects.

Molecular species	Plasma vs Serum (YM)		Plasma vs Serum (YF)		Plasma vs Serum (EM)		Plasma vs Serum (EF)	
	MFC (S/P)	*p* value	MFC (S/P)	*p* value	MFC (S/P)	*p* value	MFC (S/P)	*p* value
**Arachidonic acids (20∶4 FA) & its metabolites**								
Arachidonic acid	1.22	6.10E-05***	1.40	1.83E-04***	1.44	6.71E-03**	1.63	3.05E-04***
12-HHT	1.75	4.27E-04***	2.43	3.36E-03**	1.38	4.27E-04***	2.25	3.36E-03**
8-HETE	0.41	1.53E-03**	0.45	4.27E-03**	0.36	1.25E-02*	0.35	4.27E-03**
12-HETE	1.61	8.36E-03**	2.16	1.83E-04***	1.31	3.02E-02*	1.66	6.10E-04***
15-HETE	0.72	2.62E-03**	0.55	6.71E-03**	0.62	2.56E-02*	0.46	6.71E-03**
5,15-diHETE	0.79	1.22E-04***	0.64	5.37E-03**	0.64	1.35E-01	0.48	6.71E-03**
5,6-diHETrE	3.88	6.10E-05***	3.29	6.10E-05***	3.33	1.53E-03**	3.23	6.10E-05***
**Eicosapentaenoic acid (20∶5 FA) & its metabolites**								
Eicosapentaenoic acid	1.38	6.10E-05***	1.35	8.54E-04***	1.39	3.36E-03**	1.79	8.54E-04***
12-HEPE	1.38	1.81E-02*	1.60	2.62E-03**	1.48	2.52E-01	1.67	4.13E-02*
**Docosahexaenoic acid (22∶6 FA) & its metabolites**								
4-HDoHE	3.25	6.10E-05***	2.56	6.10E-05***	1.90	4.27E-04***	4.09	6.10E-05***
10-HDoHE	0.52	3.36E-03**	0.41	6.71E-03**	0.44	1.25E-02*	0.34	6.71E-03**
14-HDoHE	1.51	4.79E-02*	1.66	2.62E-03**	1.20	2.77E-01	2.14	6.71E-03**
20-HDoHE	0.51	3.05E-04***	0.62	1.22E-04***	0.50	5.37E-03**	0.53	5.37E-03**

(MFC (S/P), median fold change in serum/plasma; YM, young male, YF, young female; EM, elderly male; EF, elderly female; HHT, hydroxyheptadecatrienoic acid; HETE, hydroxyeicosatetraenoic acid; diHETE, dihydroxyeicosatetraenoic acid; diHETrE, dihydroxyeicosatrienoic acid; HEPE, hydroxyeicosapentaenoic acid; HDoHE, hydroxydocosahexaenoic acid).

Statistical significance was determined by Wilcoxon matched-pairs signed-rank test (*p<0.05, **p<0.01, ***p<0.001).

### Gender-associated Differences in Lipid Metabolite Levels in Blood Samples

**Figure 3:**
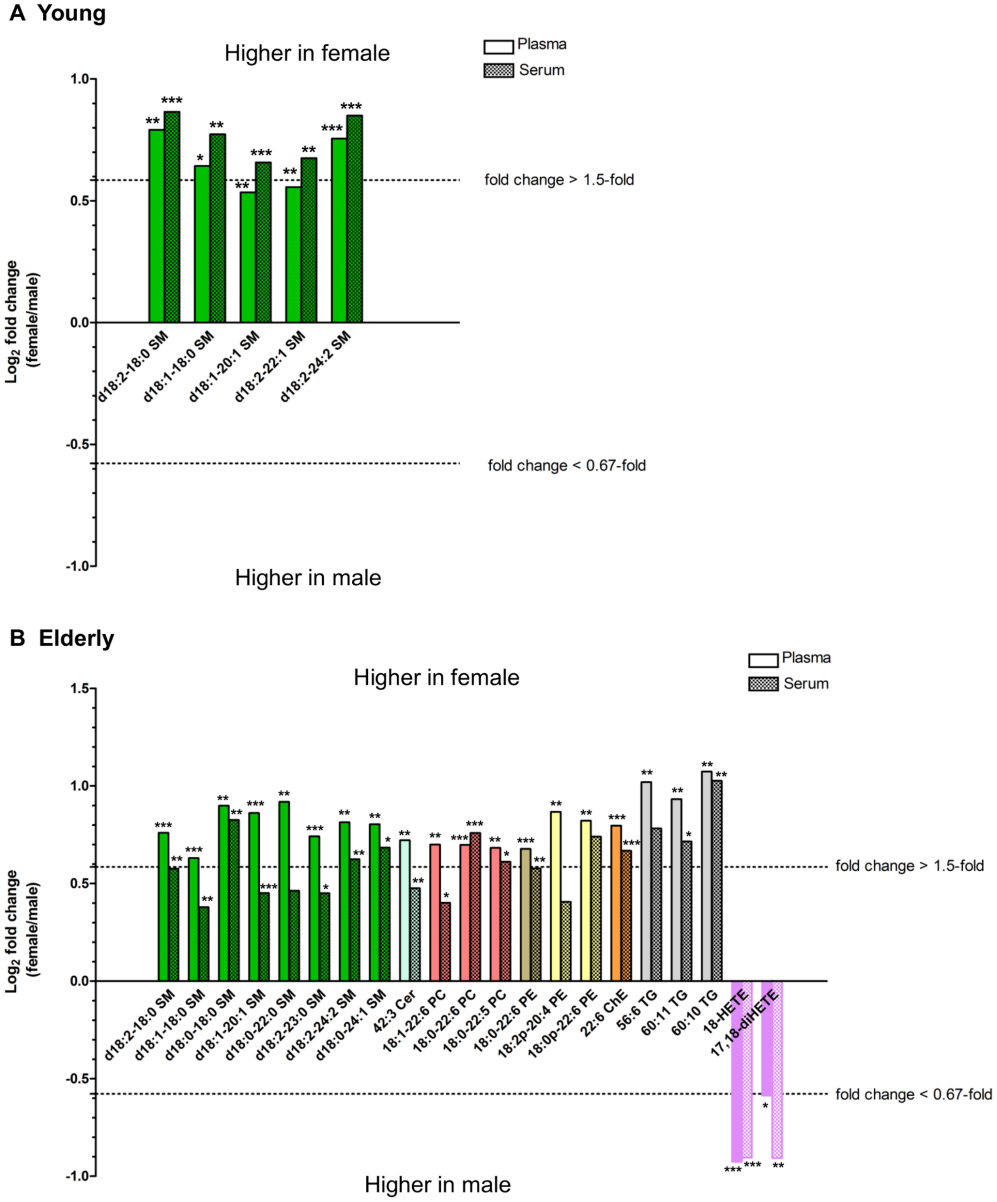
Differences in lipid metabolite levels in human blood samples between males and females. Lipid metabolites with marked fold changes (female/male >1.5 or <0.67) and *p*<0.01 values between males and females in either plasma or serum are plotted for young (A) and elderly (B) age groups. Statistical significance was determined by Mann-Whitney *U*-test (*p<0.05, **p<0.01, ***p<0.001). SM, sphingomyelin; Cer, ceramide; PC, phosphatidylcholine; PE, phosphatidylethanolamine; ChE, cholesteryl ester; TG, triacylglycerol; HETE; hydroxyeicosatetraenoic acid; diHETE, dihydroxyeicosatetraenoic acid.

**Figure 4:**
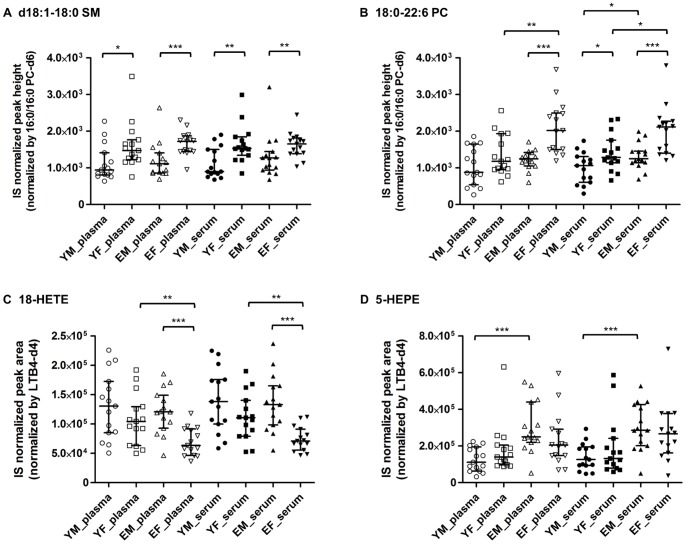
Levels of representative lipid metabolites with significant gender- and age-associated difference. (A) d18∶1–18∶0 SM, (B) 18∶0–22∶6 PC, (C) 18-HETE, (D) 5-HEPE. The graph represents medians and interquartile ranges. Statistical significance was determined by Mann-Whitney *U*-test (*p<0.05, **p<0.01, ***p<0.001). YM, young male; YF, young female; EM, elderly male; EF, elderly female. SM, sphingomyelin; PC, phosphatidylcholine; HETE, hydroxyeicosatetraenoic acid; HEPE, hydroxyeicosapentaenoic acid.

To clarify whether gender is a confounding factor for biomarker exploration studies, we investigated gender-associated differences in lipid metabolite levels in both plasma and serum samples from subjects of both age groups. Of the 253 lipid metabolites analyzed, significant gender-associated differences were observed for 16 metabolites in plasma and 20 in serum samples of the young age group, as well as for 61 metabolites in plasma and 33 in serum samples of the elderly age group (*p*<0.05; Table S4). Figure 3 summarizes the molecular species found to have marked fold changes in their levels (female/male ratio >1.5 or <0.67) and lower *p*-values (*p*<0.01) in either plasma or serum. In the young age groups (Figure 3A), the levels of SM species were markedly higher in females than in males. Differences in the levels of a representative SM species (d18∶1–18∶0 SM) among all four groups are precisely depicted in Figure 4A. In the elderly age groups (Figure 3B), not only SMs but also species of various other classes (PCs, PEs, ether PEs, Cers, ChE, TGs, and oxFA) of lipid metabolites showed markedly different levels between genders, indicating that gender-associated differences were more prominent in elderly subjects than in young subjects. Notably, in the elderly age group, the levels of five lipid metabolites containing docosahexaenoic acid (DHA), including 18∶0–22∶6 PC (Figure 4B), 22∶6 ChE, and 18∶0:22∶6 PE, were remarkably higher in females than in males.

No PUFA and oxFA metabolites were found to have significant gender-associated differences in both young and elderly age groups; however, the levels of 18-HETE (Figure 4C) and 17,18-diHETE were markedly lower in elderly females than in elderly males (Figure 3B). These changes were observed in both plasma and serum samples.

### Age-associated Differences in Lipid Metabolite Levels in Human Blood Samples

**Figure 5:**
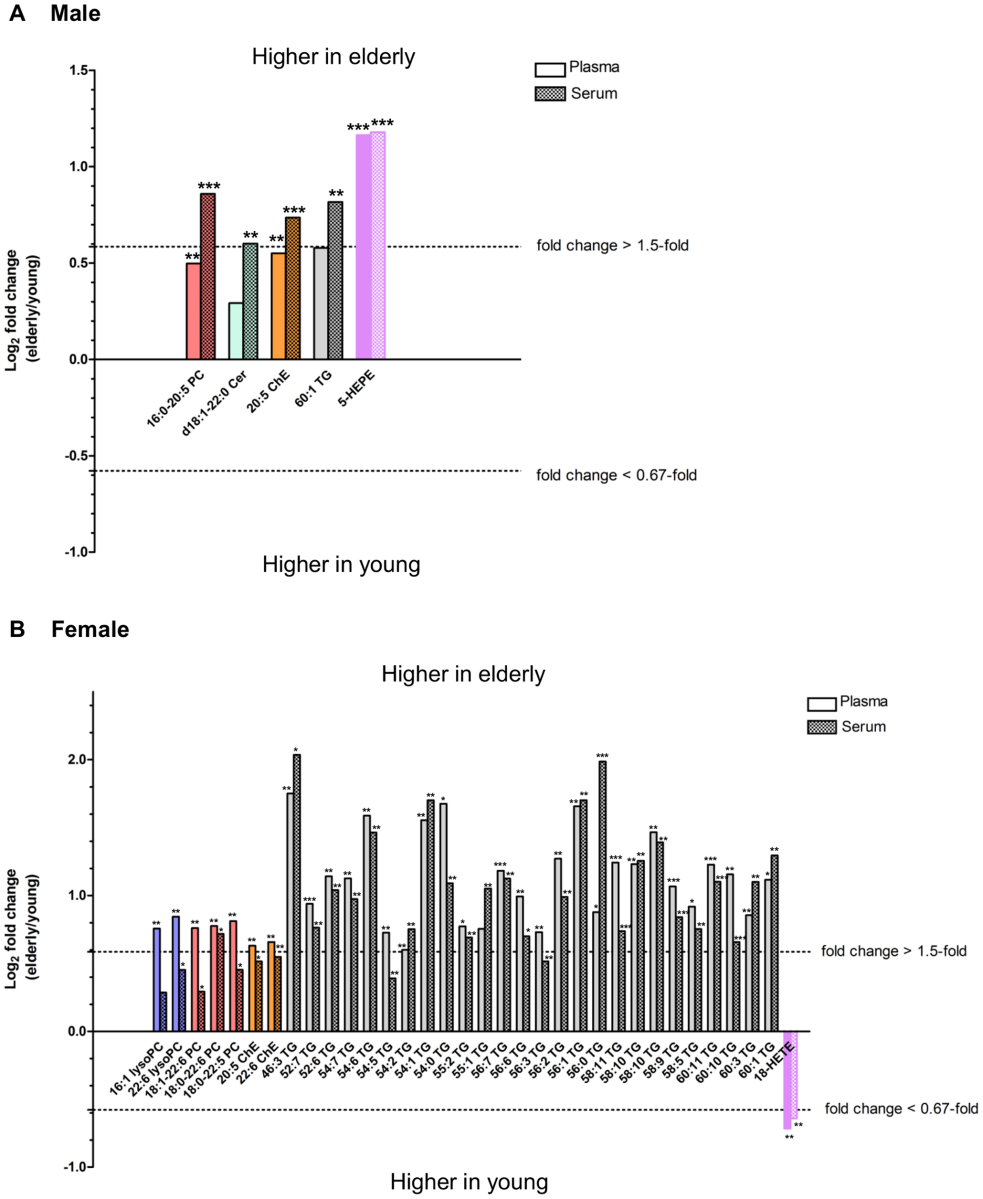
Differences in lipid metabolite levels in human blood samples between young and elderly age groups. Lipid metabolites with marked fold changes (elderly/young subjects ratio >1.5 or <0.67) and *p*<0.01 values between young and elderly age groups in either plasma or serum are plotted for male (A) and female (B) groups. Statistical significance was determined by Mann-Whitney *U*-test (*p<0.05, **p<0.01, ***p<0.001). PC, phosphatidylcholine; Cer, ceramide; ChE, cholesteryl ester; TG, triacylglycerol; HEPE, hydroxyeicosapentaenoic acid; lysoPC, lysophosphatidylcholine; HETE, hydroxyeicosatetraenoic acid.

The lipid metabolite levels were compared between the young and elderly subjects of both genders. Of the 253 metabolites, eight metabolites in plasma and 29 in serum samples of males, and 81 metabolites in plasma and 59 in serum samples of females showed significant age-associated differences (*p*<0.05; Table S5). Of these, the metabolites with marked fold changes (elderly/young ratio >1.5 or <0.67) and lower *p*-values (*p*<0.01) in either plasma or serum are shown in Figure 5. In males (Figure 5A), the levels of metabolites in the young age group were generally comparable to those in the elderly age group, with a few exceptions: one metabolite in plasma (5-hydroxyeicosapentaenoic acid [5-HEPE], Figure 4D) and five in serum (16∶0–20∶5 PC, d18∶1–22∶0 Cer, 20∶5 ChE, 60∶1 TG, 5-HEPE) were present at remarkably higher levels (fold changes >1.5; *p*<0.01) in the elderly age groups than in the young age groups.

Age-associated differences were more prominent in females than in males (Figure 5B), and many TGs were measured at significantly higher levels in elderly females than in young females in both matrices. In females (Figure 5B), the levels of 28 metabolites (two lysoPCs, three PCs, two ChEs, 20 TGs, and one oxFA) in plasma and 23 (22 TGs and one oxFA) in serum differed markedly between young and elderly age groups (fold change >1.5 or <0.67; *p*<0.01). It should be noted that female-specific age-associated changes in the levels of lysoPCs were clearly observed in plasma but were markedly less in serum (Figure 5B), suggesting that increases in the levels of lysoPCs in serum caused by the clotting process during sample preparation may obscure the original variations between the different aged groups. As for oxFAs, the levels of the cytochrome P450 (CYP) metabolite 18-HETE (Figure 4C), were remarkably lower in elderly than in young females for both matrices.

### Freeze-thawing Effect on Lipid Metabolite Stability

**Figure 6:**
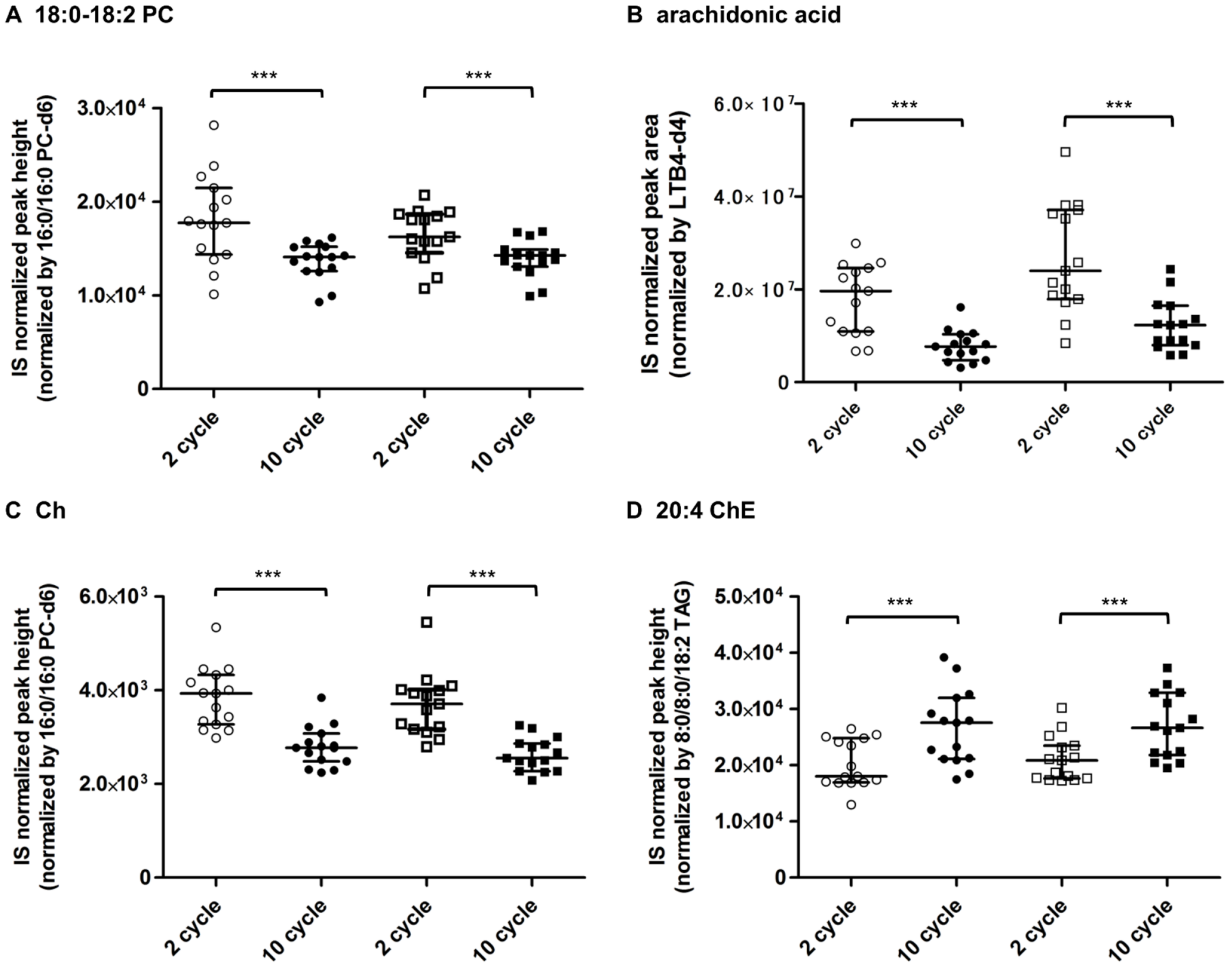
Effects of repeated freeze-thawing on lipid metabolite stability in plasma and serum of young males. Lipid metabolite levels after 10 freeze-thaw cycles were compared with those after two freeze-thaw cycles for plasma and serum samples. (A) 18∶0–18∶2 PC, (B) arachidonic acid, (C) Ch, (D) 20∶4 ChE. The graph shows medians and interquartile ranges. Statistical significance was determined by Wilcoxon matched-pairs signed-rank test (**p<0.01, ***p<0.001). PC, phosphatidylcholine; Ch, cholesterol; ChE, cholesteryl ester.

The stability of lipid metabolites was examined using 10 cycles of freezing and thawing in plasma and serum. The levels of most lipid metabolites in the samples subjected to repeated freeze-thaw cycles were greatly decreased compared with those in the normal samples (Tables S6 and S7). Representative metabolites for which marked differences were observed are shown in Figure 6. The effects of freeze-thawing were comparable between plasma and serum for almost all metabolites. For example, the levels of one major phosphoglycerolipid (18∶0–18∶2 PC) were decreased by about 79% and 88% in plasma and serum, respectively, by repeated freeze-thawing cycles (Figure 6A). In particular, PUFA and oxFAs were markedly decreased by 10 cycles of freeze-thawing (Table S7; arachidonic acid is shown in Figure 6B). Remarkably, the levels of PUFAs containing ChEs, such as 20∶4 ChE, were significantly increased by 10 cycles of freeze-thawing (Figure 6D), whereas those of free Ch as a substrate of ChEs was significantly decreased (Figure 6C).

## Discussion

We performed a comprehensive lipidomic analysis of blood from healthy adults under various conditions on sample preparation (plasma collection versus serum collection), sample selection (subjects’ age and gender) and storage (few versus more number of freeze-thaw cycles). First, our results demonstrated that, compared with serum, plasma is a more suitable matrix for exploring lipid biomarkers. We found matrix-associated differences among the levels of lysoPCs, DGs, free PUFAs, and several oxFAs, irrespective of the subjects’ backgrounds. In the preparation of serum from whole blood, the clotting process results in thrombin-stimulated platelets releasing DG and inositol 1,4,5-phosphate (IP_3_) by the degradation of phosphatidylinositol 4,5-bisphosphate (PIP_2_) via the activation of phospholipase C (PLC) [28]. Furthermore, lysoPLs and PUFA are released from membrane PLs by activated phospholipase A_2_ (PLA_2_) via IP_3_ signaling. Several oxFAs such as TXB_2_, 12-HHT, and 12-lipoxygenase products (12-HETE, 12-HEPE and 14-HDoHE) are also known to be released from activated platelets, thereby exhibiting a variety of biological effects [29]. The levels of these metabolites in serum are therefore unlikely to reflect normal biological processes in the body. Furthermore, because the clotting process is difficult to control strictly *in vitro*, it is possible that serum conceals the changes in lipid metabolism that have occurred in the body, thus obscuring variations between samples/groups. Indeed, we observed that age-associated differences in lysoPCs levels in females were less represented in serum than in plasma (Figure 4B), suggesting that the elevation of lysoPC levels in serum by the clotting process conceals the underlying age-associated differences in blood.

Mas et al. reported that the concentrations of 18-HEPE and 17-HDoHE in plasma were approximately two-fold greater than those in serum following 3 weeks of oral supplementation with n-3 fatty acids [30]. Although the metabolites detected in our study and the study by Mas and colleagues were not identical because we did not carry out a dietary intervention in our study, our results reveal, for the first time, that plasma contains higher levels of some hydroxy-fatty acids (8-HETE, 15-HETE, 10-HDoHE, and 20-HDoHE) than does serum. Degradation of these lipid metabolites in serum may occur during the clotting process. When comparing matrix-associated differences, we found, for the first time, that 5, 6-diHETrE was present at markedly (more than 3-fold) higher levels in serum than in plasma (Figure 2C, Table 2). In platelets, 5,6-epoxyeicosatrienoic acid (EET), a precursor of 5,6-diHETrE, is proposed to be a Ca^2+^ entry activator following depletion of intracellular Ca^2+^ stores caused by the activation of IP_3_ cascades [31]. This finding therefore strongly supports the hypothesis that increased levels of 5,6-diHETrE are associated with the clotting process.

Compared with our previous studies using non-fasting plasma and serum of young males and females [21], a higher number of metabolites that show matrix-associated differences was detected in fasting blood than in non-fasting blood samples. For example, significantly higher levels of lysoPCs and free PUFAs such as arachidonic acid and eicosapentaenoic acid were observed in serum than in plasma from fasting blood samples, but not from non-fasting blood samples, suggesting that fasting and non-fasting blood should be treated as different samples when exploring biomarkers in the two matrices.

Next, our results demonstrated that gender and age influence the levels of several metabolites in blood and therefore are confounding factors in exploring lipid biomarkers. Gender-associated differences highlighted that the levels of SMs are remarkably higher (fold change >1.5-fold and *p*<0.01) in females than in males irrespective of the subjects’ age (Figure 3). Moreover, body mass index (BMI) was found to be significantly higher in females than in males (Table 1), raising the possibility that differences in the levels of SMs are associated with BMI rather than with gender. However, gender-associated differences in SMs were observed even when subgroups of males and females with comparable BMI levels were compared (data not shown), indicating that the influence of BMI on SM levels is marginal. It was reported in a prospective study that the levels of SM species in plasma were significantly lower (*p*<0.05) in AD subjects than in cognitively normal control subjects [14], and are thus of interest as AD biomarkers. Our results suggest that gender-associated differences in SM levels should be paid attention for their application as biomarkers in a clinical setting in the future.

Previous research suggests that estrogen may be involved in the regulation of SM metabolism [32]. It has been also reported that young children with low levels of sex hormones exhibit gender-associated differences in plasma SM levels [23]. The potential role of estrogen in SM metabolism therefore remains controversial. In the present study, we show that SMs are present at higher levels in elderly females than in elderly males, and at comparable levels in young and elderly females. This finding was observed despite the levels of estrogen being markedly decreased in postmenopausal elderly females, usually lower even than in elderly males [33]. This result suggests that factors other than estrogen are probably responsible for gender-associated differences in SM levels. The exact mechanisms of the regulation of SM metabolism have not been elucidated thus far.

Upon further investigation of fatty acyl chains of PLs, our analysis revealed that DHA-containing lipid metabolites such as 18∶1–22∶6 PC and 18∶0–22∶6 PC (Figure 4B) showed both gender- and age-associated differences in matrices of elderly females (Figures 3 and 5). A recent study suggested that estradiol influence the activity of enzymes that are involved in the synthesis of DHA in rats [34]. Our observations suggest that the change in estrogen secretion following menopause may affect DHA metabolism. Although their mechanisms involved in this potential connection between estrogen levels and DHA metabolism remain to be elucidated, DHA metabolite levels may be influenced by both ageing and genders.

Age-associated differences in lipid metabolite levels were more prevalent in females than in males. Many TGs, in particular, showed age-associated differences in females but not in males (Figure 5). This observation suggests that the decrease in estrogen secretion following menopause [33] affects the metabolism of several lipoproteins, resulting in an increase in the levels of TGs in the elderly. Estrogen may be involved in regulation of the metabolism of lipoproteins such as low-density lipoprotein [35]. As for males, a 5-lipoxygenase (5-LOX) product, 5-HEPE, was significantly higher in elderly than in young subjects, and a similar trend was observed between young and elderly females (Figure 4D). The bioactive lipid 5-HEPE is known to be a potent agonist of GPR119 and enhances glucose-dependent insulin secretion [36], suggesting the molecule as a possible biomarker candidate for diabetes. Although the mechanisms regulating age-associated increases in 5-HEPE are unknown, Schuchardt et al. reported that serum levels of EPA-derived oxylipins, including 5-HEPE, strongly correlate with the EPA content of the erythrocyte membrane, and thus with the availability of the substrate EPA [37]. Furthermore, results from a dietary intervention study suggest that elderly subjects have a greater capacity to incorporate dietary EPA into plasma phospholipids and cells than younger subjects do [38], [39]. Taken together, these results indicate that increased EPA availability in elderly subjects may result in higher 5-HEPE levels in elderly males than in young males. Indeed, EPA-containing lipids such as 16∶0–20∶5 PC and 20∶5 ChE showed age-associated increases in males (Figure 5).

One major ceramide species in plasma, d18∶1–22∶0 Cer, also exhibited age-associated increases in the serum of male subjects. Plasma ceramide is most likely synthesized in the liver by ceramide synthases from sphinganine and acyl coenzyme A, after which it is incorporated into lipoproteins and transported into plasma [40]. The augmented levels of this species in elderly males may be linked with the biological function of ceramides as signaling molecules capable of regulating vital cellular functions, including apoptosis, cell growth, differentiation, senescence, diabetes, insulin resistance, inflammation, neurodegenerative disorders, and atherosclerosis [41]. It is interesting to note that two metabolites with opposing roles, 5-HEPE (insulin secretion) and d18∶1–22∶0 Cer (insulin resistance), are concurrently increased with age in healthy males.

Based on the results of the lipidomic analysis of serum and plasma samples subjected to repeated freeze-thaw cycles compared to normal samples, we recommend that plasma and serum samples should be aliquoted after their collection, and repeated freeze-thaw cycles should be avoided. Most lipid metabolite levels were significantly decreased by repeated freeze-thaw cycles in both plasma and serum (Tables S6 and S7, Figure 5A–C), suggesting that lipid metabolites are degraded or metabolized by various lipases such as phospholipase A_2_ (PLA_2_) during the process of freeze-thawing. However, PUFA-esterified ChEs were significantly increased by repeated freeze-thaw cycles (Figure 5D). Because lecithin-cholesterol acyltransferase (LCAT) is primarily responsible for producing PUFA-esterified ChEs such as 20∶4 ChE and 22∶6 ChE [42], these results suggest that the LCAT-mediated reaction in which the acyl chain at the *sn*-2 position is transferred from PLs to Ch is activated during the freeze-thaw process in each matrix.

## Conclusions

Lipidomics could be a promising new approach for the identification of new biomarkers for monitoring or predicting disease states and/or drug responsiveness. In this study, we obtained fundamental information on sample selection and handling procedures applied to measuring lipid metabolites in blood, which is important in ensuring the quality of biomarker discovery and qualification processes. Our results suggest that plasma (rather than serum), subjected to a minimum number of freeze-thaw cycles, is suitable for obtaining reliable measurements of lipid levels reflecting basal, physiological levels. Notably, repeated freeze-thawing is a detrimental procedure resulting in a reduction in the levels of the majority of lipid metabolites, consequently leading to erroneous data. In general, ideal metabolomic biomarkers are those whose levels are drastically modulated by disease or drugs so as to overcome any variations in subjects’ background levels. Otherwise, we should keep in mind that age and gender are confounding factors when measuring several metabolites. Our current data showing gender- and age-associated differences in each lipid metabolite are key pieces of information to be considered when selecting candidate biomarkers for further validation. It is conceivable that lifestyle (e.g., drinking, smoking and dietary habits) and ethnicity may also affect metabolite levels in blood, and further study is needed to examine these effects.

## Supporting Information

Table S1 **Dataset of internal standard (IS)-normalized peak hight of phosphoglycerolipids, sphingolipids and neutral lipids in human blood.**
(XLSX)

Table S2 **Dataset of internal standard (IS)-normalized peak area of polyunsaturated fatty acids and their oxidative metabolites.**
(XLSX)

Table S3 **Matrix-associated differences (fold changes and statistical analysis) in the levels of lipid metabolites.**
(XLSX)

Table S4 **Gender-associated differences (fold changes and statistical analysis) in the levels of lipid metabolites.**
(XLSX)

Table S5 **Age-associated differences (fold changes and statistical analysis) in the levels of lipid metabolites.**
(XLSX)

Table S6 **Effects of freeze-thawing on phosphoglycerolipids, sphingolipids and neutral lipids stability.**
(XLSX)

Table S7 **Effects of freeze-thawing on polyunsaturated fatty acids and their oxidative metabolites stability.**
(XLSX)
